# Can invitation systems increase participation in preventive health screening among adolescents?—an evaluation of a state-wide intervention in Germany using a difference-in-differences analysis of claims data

**DOI:** 10.1093/eurpub/ckaf026

**Published:** 2025-03-07

**Authors:** Iryna Iashchenko, Wiebke Schüttig, Philip Bammert, Udo Schneider, Jacob Spallek, Petra Rattay, Sven Schneider, Irene Moor, Claudia R Pischke, Leonie Sundmacher

**Affiliations:** Chair of Health Economics, TUM School of Medicine and Health, Technical University of Munich, Munich, Germany; Munich Center for Health Economics and Policy, Munich, Germany; Chair of Health Economics, TUM School of Medicine and Health, Technical University of Munich, Munich, Germany; Munich Center for Health Economics and Policy, Munich, Germany; Chair of Health Economics, TUM School of Medicine and Health, Technical University of Munich, Munich, Germany; Munich Center for Health Economics and Policy, Munich, Germany; Techniker Krankenkasse, Hamburg, Germany; Department of Public Health, Brandenburg University of Technology Cottbus-Senftenberg, Senftenberg, Germany; Lusatian Centre for Digital Public Health, Brandenburg University of Technology Cottbus-Senftenberg, Senftenberg, Germany; Department of Epidemiology and Health Monitoring, Robert Koch Institute, Berlin, Germany; Center for Preventive Medicine and Digital Health (CPD), Medical Faculty Mannheim, Heidelberg University, Mannheim, Germany; Institute of Medical Sociology, Interdisciplinary Center for Health Sciences, Medical Faculty, Martin-Luther-University Halle-Wittenberg, Halle (Saale), Germany; Institute of Medical Sociology, Centre for Health and Society, Medical Faculty, Heinrich-Heine-University, Duesseldorf, Germany; Chair of Health Economics, TUM School of Medicine and Health, Technical University of Munich, Munich, Germany; Munich Center for Health Economics and Policy, Munich, Germany

## Abstract

In Germany, routine screenings are used to monitor the health and development of children and adolescents, enabling timely discovery and treatment of health issues. One such screening, called J1, is recommended for adolescents aged 12–14 years, but participation is only 43%. In the state of Bavaria, a lack of awareness is the main reason cited for not attending. ‘Your Ticket to J1’ was an invitation system implemented across the state to inform adolescents about the screening. Our study investigated whether this intervention increased J1 participation and if its effects varied by family socioeconomic position (SEP). We used pseudonymized data from a large statutory health insurer from 2016 to 2018 and containing 267 650 observations. To investigate the effect of the intervention, we employed a difference-in-differences analysis at the individual level. Assuming parallel trends at the state level, we compared J1 participation rates between Bavaria and other German states before and after the intervention. We additionally stratified analyses by SEP. The intervention led to an increase in J1 participation by about 1%. In the stratified regressions, the effect size was larger for children from families with a lower SEP. J1 participation increased by about 4% among adolescents whose primary insured parent had the lowest occupational status. A state-wide invitation system had a small but statistically significant positive impact on J1 participation and might reduce socioeconomic inequities in healthcare utilization. Informing adolescents about J1 appears to increase participation, particularly among those from families with a lower SEP.

## Introduction

Socioeconomic inequities in the health of children and adolescents are a major concern in modern societies [1, 2]. Numerous studies have shown that early-life exposure to poorer socioeconomic conditions is associated with poorer health in children [3], leading to long-lasting health effects [4, 5]. Socioeconomic inequities also exist in healthcare utilization among children. International evidence suggests that children from families with a lower socioeconomic position (SEP) or those living in socially deprived areas use health services less frequently compared to children from high SEP families [6–8]. This phenomenon can be observed even in countries with universal healthcare coverage [9, 10]. The KiGGS Study indicated that children from low SEP families in Germany use preventive healthcare services less often than their high SEP counterparts [9].

In Germany, routine screenings are used to monitor the health and development of children and adolescents, enabling timely discovery and treatment of health issues. These screenings begin with U0 (*Untersuchung 0*, or ‘Examination 0’), which occurs shortly before birth, followed by a series of standardized examinations known as U1 through U9, each corresponding to age milestones in a child’s early development. The screenings continue with U10 and U11, which focus on older children. The *Jugenduntersuchung 1* (J1), or ‘Youth Examination 1’, is a preventive health examination recommended for adolescents in Germany between the ages of 12 and 14 years [11]. J1 includes an assessment of physical and mental health, growth and development, and a review of immunization records for any missing vaccinations. Additionally, the physician discusses various health topics with the adolescents, such as puberty issues, alcohol and drug consumption, family problems, school performance, and career aspirations [12].

Attending J1 between the ages of 12 and 14 might also be important for preventing addictions, ensuring the correct use of contraception, and reducing the risk of sexually transmitted infections (STIs). In Germany, about 8% of adolescents around the age of 13 and 30% of those around the age of 15 have tried smoking, and 15.5% of 15-year-olds have tried cannabis. About 70% of adolescents have tried alcohol by the age of 15, with 40% having participated in binge drinking [13]. Smoking is more prevalent among adolescents from disadvantaged socioeconomic backgrounds [14]. More than half of adolescents have their first sexual experience between the ages of 14 and 17 years [15].

Statutory health insurance in Germany covers the full cost of preventive examinations U1–U9 and J1. Nevertheless, findings from the KiGGS study suggest that adolescents from families with a lower SEP and those with a family history of migration are less likely to attend them [16, 17]. This suggests the presence of socioeconomic inequities in health care utilization. Moreover, in contrast to the well-established U1–U9 examinations for younger children, which have attendance rates exceeding 90%, the average participation rate for J1 in Germany is about 43% [11]. Participation also varies by gender, with girls being less likely to attend, and by city size, with children from smaller cities having lower attendance rates (ibid.).

The reasons for low participation in J1 are not completely understood. One potential explanation is a lack of awareness of the examination. The highly attended U1–U9 examinations are scheduled between birth and 6 years of age. In a recent survey of parents and adolescents in Bavaria, one of the most frequently cited reasons for not attending J1 was unawareness of it [18]. As children grow older, such examinations become less frequent and parents may lose track of upcoming appointments. Other reasons cited for non-participation have been fears of the examination and a lack of time [19].

Several German states have introduced regional policies to motivate adolescents to participate in J1, a summary of which is presented in Table S1. The first J1 invitation systems in Germany started in 2008 in Rhineland-Palatinate and Brandenburg, resulting in a significant increase in J1 participation [20, 21]. For example, in Brandenburg, participation increased by 1.8% in the first year after the intervention and by an additional 10% in the following year (ibid.). The pilot information campaign in Mecklenburg-Western Pomerania in 2011 raised J1 participation rates by 24% (between 14% and 33%, depending on the region) [22]. The short information campaign in Baden Wuerttemberg in 2015 also reported promising results [23]. Furthermore, the supra-regional campaign ‘Your Next Top Check-up J1’ in 2012, organized by the national and seven regional associations of statutory health insurance physicians, disseminated invitations regionally through their own communication channels but not personalized. A recent evaluation of J1 participation rates showed that participation was generally higher in states with existing invitation systems [24].

One such system, ‘Your Ticket to J1’, was introduced in Bavaria in 2017and financed by the Bavarian State Ministry of Health and Care [25]. It consists of an informational flyer designed as an invitation ticket and distributed during the routine immunization pass review in sixth grade. During the review adolescents receive the flyer along with vaccine recommendations. The flyer is targeted at adolescents aged 12–14 years and features illustrated characters who explain what attending J1 involves, including the questions asked and the examinations performed. The ticket on the flyer was designed to resemble an entry ticket for an event, such as a concert, but presenting it was not required to access the examination.

A pilot study for ‘Your Ticket to J1’ was organized in autumn 2016 in four districts in Bavaria, with two serving as intervention districts and two as controls. All 12- to 14-year-olds residing in the intervention districts received the flyer by standard mail. In one of the intervention districts, sixth-grade students additionally received the flyer at schools during the review of their immunization passes. An evaluation of this pilot intervention showed that participation rates increased by 9.1% and 16% in intervention districts compared to the control districts [25]. A larger rise in participation was observed in the intervention district in which students additionally received the ticket at school. ‘Your Ticket to J1’ was introduced throughout Bavaria in mid-2017 as part of the review of immunization passes at schools among sixth-graders (ibid.)

The aims of the present analysis were to investigate whether the intervention ‘Your Ticket to J1’ was effective in increasing participation in J1 in routine care, and if the effects varied by family SEP.

## Methods

### Study design

The invitation system ‘Your Ticket to J1’ can be considered a natural experiment because it was introduced in Bavaria at an arbitrary point in time and was not implemented in other German states. We therefore employed a quasi-experimental difference-in-differences (DiD) design to evaluate the effects of the intervention [26]. Several states with similar state-wide policies in the past (Rhineland-Palatinate, Brandenburg, Mecklenburg-Western Pomerania, Baden-Wuerttemberg, see Table S1) were excluded from the analysis. Assuming parallel trends at the state level, we compared the J1 participation of 13- and 14-year-olds (birth years 2003, 2004 and 2005, 2006) between Bavaria and the other German states before and after the intervention (years 2016 and 2018). To investigate differences in effects by SEP, we conducted the analysis additionally for different SEP strata.

### Data

We use pseudonymized claims data from the statutory health insurer Techniker Krankenkasse (TK) from 2016 to 2018. TK is the largest health insurance provider in Germany, covering about 13% of the population as of 2022 [27]. The data included individual-level information on ambulatory care and sociodemographic background. We included patients who were insured in all quarters of the respective observation periods. A special feature of the dataset was the ability to link a child’s data to the parent through whom the child was insured (i.e. the primary insured parent). This linkage allowed us to include socioeconomic and sociodemographic variables of the family, such as the parent’s education, occupation, and citizenship. All further references to parents in this study are to the primary insured parent. We also included two regional covariates—density of paediatricians and regional household income at the community level—which we obtained from the INKAR database of the Federal Institute for Research on Building, Urban Affairs and Spatial Development [28].

### Variables

#### Outcome variable

The outcome variable was the participation in J1 at the individual level, coded as 1 for participation and 0 for non-participation in a given year. The screening was identified in the claims data through the doctor’s fee scale ‘01720’.

#### Control variables

We included the adolescent’s gender (female: 1; male: 0) and the citizenship of the primary insured parent (German: 1; other: 0) as control variables. Additionally, to control for access to healthcare, we included the density of paediatricians at the district level (paediatricians per 10 000 children aged up to 15 years). To account for regional deprivation/wealthiness, we included regional household income at the district level (average household income in euros). Additionally, we included state fixed effects to control for unobservable factors within the states. In choosing these variables, we relied on previous studies suggesting they could affect the utilization of J1 among adolescents [11, 16].

#### SEP variables

The SEP variables in this study, namely parental education and occupation, were inferred from the parental employment classification code available in the claims data. We grouped educational attainment into three levels: low (1: no professional qualifications), average (2: average professional qualifications, such as vocational training), and high (3: university level). Higher levels of educational attainment are associated with higher salary expectations and greater health literacy in Germany [29, 30]. The categorization of occupation groups was derived from the parental employment classification code and consists of four occupation status levels: assistant job (1: e.g. au-pair), skilled employee (2: e.g. plumber), specialist: (3: e.g. nursery-school teacher), expert (4: e.g. physician). These four occupation groups vary in the level of education and complexity of professional requirements. Similar to education, higher occupation status corresponds to higher salary expectations. If the included parent had multiple entries with different values for education and occupation, we selected the highest levels attained.

### Statistical analysis

We aimed to evaluate the effect of introducing the J1 invitation system in Bavaria on the J1 participation rates among 13- and 14-year-olds. Using a DiD approach, we estimated the following equation:


$$Y_{\mathrm{ist}}=\beta_{0}+\;\beta_{1}{\mathrm{Time}}_{t}+\;{\beta_{2}\mathrm{Treatment}}_{s\;}+\;\beta_{3}{\mathrm{Policy}}_{st}+\;{\beta_{4}X}_{\mathrm{ist}}+\delta_{s\;}+\;e_{\mathrm{ist}},$$

where $Y_{\mathrm{ist}}$ is the participation status in J1 by 13- and 14-year-olds in year *i*, state *s*, and time *t*, ${\mathrm{Time}}_{t\;}$ is a time dummy (1: year 2018, 0: year 2016), ${\mathrm{Treatment}}_{s}$ is a treatment group dummy (1: Bavaria, 0: other states), ${\mathrm{Policy}}_{st}$ is an interaction term for time and treatment (intervention) dummy (1: Bavaria in 2018, 0: other variations), $X_{\mathrm{ist}}$ represents explanatory covariates to account for differences across individuals and states, $\delta_{s\;}$ are state fixed effects, and $e_{\mathrm{ist}}$ are heteroscedasticity robust standard errors clustered at the state level. The coefficient $\beta_{0}$ shows the baseline average outcome of the control group before the intervention, $\beta_{1}$ shows the difference between the average outcome of the control group before and after treatment, $\beta_{2}$ measures the difference between the two groups before treatment, and $\beta_{3}$ measures the difference in changes over time and reflects the effect of the policy.

We started with the classic minimal DiD model (Model 1). In Model 2, we added individual-level covariates, SEP variables, and regional variables. For Model 2, we excluded observations with missing values in any of the explanatory covariates. About 13% of adolescents could not be matched to a parent, and about 10% of the matched parents had missing values in the employment classification code, which could not be completed with other information. To evaluate the effect of the intervention in different SEP strata, we performed stratified regressions by occupation and education.

## Results

The dataset included a total of 267 650 adolescents. Table 1 provides descriptive statistics of the population before the intervention. We only used observations without any missing values. Both groups were similar in terms of gender, parental occupation status, educational attainment, and citizenship. On average, however, the regional household income was higher and the density of paediatricians was lower in Bavaria than in the control regions. A larger percentage of parents in the intervention group had a higher occupation status and education levels.

**Table 1.** Descriptive statistics before policy introduction, year 2016

	Control			Intervention		
	Mean	SD	*N*	Mean	SD	*N*
Female	0.49	0.50	86.085	0.50	0.50	15.343
Parental German citizenship	0.93	0.25	86.085	0.92	0.27	15.343
Regional household income	1766.90	185.94	86.085	2073.56	289.72	15.343
Density of paediatricians	5.16	1.22	86.085	4.82	2.04	15.343
Parental occupational status	%		N	%		N
1 low	6.99		6.020	5.02		770
2	44.40		38.226	37.48		5.751
3	20.35		17.517	23.01		3.530
4 high	28.25		24.322	34.49		5.292
Parental educational attainment						
1 low	4.17		3.590	3.53		542
2	59.86		51.534	53.22		8.166
3 high	35.97		30.961	43.24		6.635

Note: The range of the included variables: Female: 0–1, Occupation: 1–4; Education: 1–3; German: 0–1; Regional household income: 1292–2844; Density of paediatricians: 1.34–13.52.

### Assessment of the parallel trends assumption

**Figure 1.**
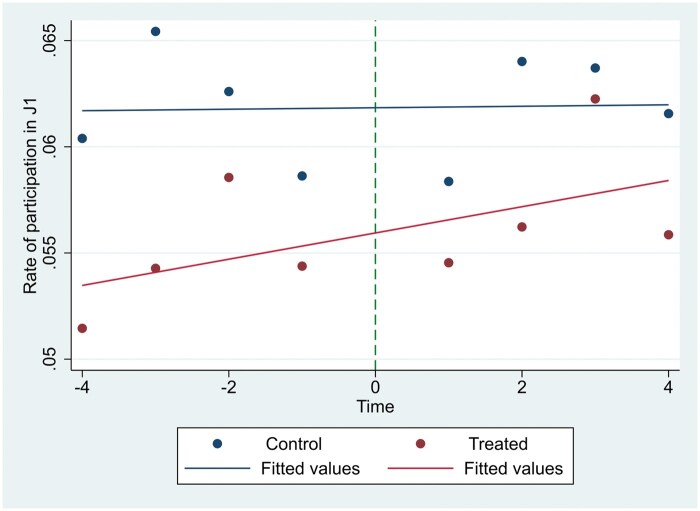
Graphical depiction of linear trend lines of J1 participation in the intervention and control groups by quarter.

We performed a graphical assessment of the parallel trends assumption. Figure 1 shows the participation rate in J1 plotted against the time variable in the four quarters before and the four quarters after the intervention. A small vertical shift is noticeable in the participation rate estimates in the intervention group after the introduction of the invitation system. The trends before the treatment appear relatively parallel, with the trend line in the intervention group showing a slight upward trend after the invitation system was introduced.

The invitation system had a small but positive significant effect on participation in J1 for 13- and 14-year-olds. Table 2 shows the results for Models 1–2. The participation rate increased by about 1% in the treatment group compared to the control group. This effect was significant in both models. The occupational status and educational attainment of the primary insured parent were positively correlated with an adolescent’s participation in J1. Additional variables that were positively correlated with J1 participation included regional household income and the density of paediatricians. Finally, being female was negatively correlated with participation in J1.

**Table 2.** Regression estimates for participation in the J1 examination for Models 1 and 2

	Model 1	Model 2
Time	0.0007 (0.0019)	−0.0039 (0.0026)
Treatment	−0.0252*** (0.0010)	−0.0324^***^ (0.0031)
Policy	0.0097^***^ (0.0019)	0.0101^***^ (0.0015)
State fixed effects	**✓**	**✓**
Female		−0.0079^***^ (0.0016)
Parental occupational status (ref. 1—low)		
2		0.0368^***^ (0.0021)
3		0.0465^***^ (0.0029)
4		0.0460^***^ (0.0044)
Parental educational attainment (ref. 1—low)		
2		0.0240^***^ (0.0046)
3		0.0364^***^ (0.0049)
Parental German citizenship		0.0067 (0.0039)
Regional household income		0.0000* (0.0000)
Density of paediatricians		0.0044^**^ (0.0010)
C	0.2436^***^ (0.0010)	0.1105^**^ (0.0259)

*N*	267650	206667
*R* ^2^	0.001	0.003
adj. *R*^2^	0.001	0.003

Standard errors in parentheses.

* *P *<* *.05. **P <* *.01. ***P <* *.001.

Table 3 shows the estimates from the stratified regressions by occupational status. The effect size was larger for adolescents whose primary insured parent had a lower occupational status, showing an increase in J1 participation of about 4.3% in group 1 compared to 1.7% in group 3 and 0.6% in group 4. Table S2 shows the estimates from the stratified regressions by parental education. The effect size was slightly larger for children whose primary insured parent had lower levels of education, showing an increase of about 1.8% in group 1 compared to 1.5% in group 3.

**Table 3.** Stratified regression estimates of participation rates in J1 by primary insured parent’s occupational status

Occupation	1 lowest	2	3	4 highest
Time	−0.0093 (0.0080)	0.0000 (0.0032)	−0.0007 (0.0043)	−0.0001 (0.0024)
Treatment	−0.0378^***^ (0.0039)	−0.0244^***^ (0.0016)	−0.0301^***^ (0.0021)	−0.0275^***^ (0.0012)
Policy	0.0425^***^ (0.0080)	0.0043 (0.0032)	0.0168^**^ (0.0043)	0.0065* (0.0024)
State fixed effects	**✓**	**✓**	**✓**	**✓**
C	0.2053^***^ (0.0039)	0.2452^***^ (0.0016)	0.2578^***^ (0.0021)	0.2650^***^ (0.0012)

*N*	13635	90178	42380	60474
*R* ^2^	0.002	0.001	0.001	0.001
adj*. R*^2^	0.001	0.001	0.001	0.001

Standard errors in parentheses.

* *P *<* *.05. **P <* *.01. ***P <* *.001.

## Discussion

Using a quasi-experimental design, we assessed the effects of an invitation system for a preventive screening examination (J1) for adolescents. We found that the intervention had a small but significant positive impact on J1 participation with a larger effect observed among adolescents from families with a lower SEP. At the individual level, higher occupation status, and higher educational attainment of the primary insured parent were positively correlated with J1 participation. At the regional level, household income and the density of paediatricians were positively correlated with J1 participation. In contrast, being female was negatively correlated with participation in J1. Overall, these findings suggest the presence of socioeconomic disparities in J1 participation. Previous studies investigating factors related to J1 participation have reported mostly similar findings [16, 17].

Similar results have been observed for invitation systems introduced in other German states [21–24], with effect sizes ranging from 1% and 24%. These variations may be attributed to differences in policy designs, data sources and study designs. Recent international evidence from a large review also showed that reminders generally improve the utilization of preventive healthcare services [31]. The 1% increase in the participation rate found in our study might seem modest, but it translates to a large number in absolute terms: with ∼1.5 million adolescents aged 13 and 14 years in Germany [32], a 1% increase in J1 participation means ∼15 000 additional examinations per year. Therefore, further efforts should be made to inform adolescents about J1 and increase their awareness of its importance.

To our knowledge, this is the first study to investigate the effect of an invitation system on participation in the J1 examination by SEP. Our findings demonstrate that the participation rate for children from lower SEP families, as measured by the occupation of the primary insured parent, increased by about 4%, whereas for children from higher SEP families, the increase was only 1%. Similarly, the participation rate increase was 0.5% higher in the group of adolescents from families with lower educational attainment. A potential explanation for this finding could be that adolescents and parents from higher SEP families were better informed about the screening program even before the intervention compared to families with low SEP. The invitation system targeted adolescents independently of their SEP background and showed stronger effects among more socioeconomically disadvantaged children. This suggests that similarly designed invitation systems have the potential to reduce socioeconomic inequity in healthcare utilization among adolescents. Earlier studies have shown similar results for the standardized childhood examinations in Germany [20, 21, 33].

Timely participation in J1 is highly relevant from a preventive health perspective because it enables early detection of adverse physical or mental conditions and allows for catching up on missing immunizations. For example, although immunization rates are quite high during early childhood in Germany, the number of adolescents receiving booster immunizations during their teenage years is comparatively low [34]. Furthermore, only 47% of 15-year-old girls received human papillomavirus (HPV) vaccinations in Germany in 2019 [35]. Some studies have shown a positive correlation between J1 participation and HPV immunizations in adolescents [24, 36]. Promoting awareness of HPV immunization during J1 screening is considered to be a potentially effective way to increase HPV vaccination rates [37].

We found that paediatrician density was positively significantly correlated with participation in J1. This suggests that better access to healthcare plays a role in the utilization of J1. This is an important finding for policy makers because improving access to healthcare for children, especially those from more socially disadvantaged families, has the potential to reduce socioeconomic inequities in healthcare use.

The main strength of our study is its use of claims data, which provide objective records of healthcare utilization and individual variables. Furthermore, we were able to link adolescents to their primary insured parent in the dataset, allowing us to evaluate the effects of the intervention based on an approximation of the socioeconomic background of the family. Our study has several limitations. First, this study is not an intervention study. We do not know the actual treatment status and therefore rely on the assumption that all adolescents in the sixth grade in Bavaria received the invitation to J1 at school in 2017. It is possible, however, that the receipt of the invitation was not universal. Second, certain age discrepancies at the time of receiving the ticket are possible. We assume that most of the sixth graders are 12 or 13 years old, but it is also possible that a sixth grader was 11 years old when he or she received the ticket and, therefore, would not be included in the analyses. These two limitations could lead to a potential underestimation of the actual treatment effect. Third, an important methodological limitation was our inability to perform a placebo test due to a lack of data before 2016. For the same reason, we could not analyse a long-term trend in the participation rates in J1 before the intervention. Fourth, we use claims data from only one health insurance provider, which covers about 13% of the population in Germany. We cannot exclude the possibility that these data might not be representative of the population of Germany as a whole. Fifth, claims data has its own limitations, such as a lack of detailed sociographic information about patients, partially incomplete employment classification codes, data delays, and the constraint of quarterly reporting in the outpatient sector. Sixth, we could only infer the socioeconomic variables of the parent through whom the adolescent was insured and were not able to observe the other parent in the data, potentially leading to an incorrect classification of the family SEP. However, we assume that the SEP of the other parent correlates with the SEP of the included parent due to spousal assortative matching [38].

It is also important to note that the participation rates we measured do not represent the overall participation rates in J1. This is because our dataset included only 13- and 14-year-olds while J1 participation is generally possible between the ages of 12 and 15 years. We focused on this narrower age range to ensure our evaluation reflected the effect of the invitation system without creating an overlap in the populations before and after the intervention. Furthermore, participation rates in J1 were lower in Bavaria compared to the other states both before and after the intervention. This disparity is well-documented and can be attributed to regional differences between the western and the eastern German states [11]. Finally, we evaluated the immediate effect of the intervention one year after the introduction of the invitation system. Previous research indicates that the immediate effect of a similar policy was initially low but increased substantially in the following year [21].

In conclusion, our findings indicate that an invitation system for the preventive examination J1 in Germany had a modest positive effect on participation among 13- and 14-year-olds, with stronger effects observed among adolescents from families with a lower SEP. Invitation flyers with a similar design and distribution method (e.g. via schools and paediatricians) have the potential to effectively reach the target groups. Further research is needed to investigate the long-term effects of the intervention and to assess whether other distribution methods could improve its effectiveness. Additionally, examining whether targeting multiple recipients of the policy message (e.g. adolescents and parents) further increases participation would be beneficial.

## Supplementary Material

ckaf026_Supplementary_Data

## Data Availability

The data that support the findings of this study were provided by the statutory health insurance Techniker Krankenkasse and are not publicly available. Restrictions apply to the availability of these data, which were used under licence for the current study (DFG FOR 2723). Data may be available upon reasonable request and with permission of the Techniker Krankenkasse and its supervisory authority.
