# Supplementary material for: Can invitation systems increase participation in preventive health screening among adolescents?—an evaluation of a state-wide intervention in Germany using a difference-in-differences analysis of claims data
Source: Eur J Public Health. 2025 Mar 7;35(2):312–8. doi: 10.1093/eurpub/ckaf026 (PMC11967887; doi:10.1093/eurpub/ckaf026)
Supplement: ckaf026_Supplementary_Data [file ckaf026_supplementary_data.zip › ckaf026_Supplementary_Data/ejph-2024-11-om-0843-File004.docx]

Table S1. Regional policies aimed at motivating adolescents to participate in J1 in Germany as of 2022.

| State | Type of policy | When introduced |
| --- | --- | --- |
| Rhineland-Palatinate | Invitation system (statewide) | 2008, ongoing |
| Brandenburg | Invitation and feedback system (statewide) | 2008, ongoing |
| Mecklenburg-Western Pomerania | Flyer and letter by standard mail (statewide) | Pilot study in 2011  Statewide since 2012, ongoing |
| Baden-Wuerttemberg | Invitation at school and letter by standard mail (statewide) | 2015 |
| National Association of Statutory Health Insurance Physicians and seven regional Associations of Statutory Health Insurance Physicians (North Rhine, Schleswig-Holstein, Hamburg, Brandenburg, Baden-Wuerttemberg, Mecklenburg-Western Pomerania and Saxony-Anhalt) | Video and flyer “Your next top Check-up J1” at the physician’s office, not personalized | 2012 |
| Bavaria | Invitation system involving flyer “Your Ticket to J1”, video and website^[[1]](#footnote-1)^ | Pilot study in 2016,  Statewide in 2017, ongoing |

1. The website was available at the beginning of the invitation program but is not further supported as of January 2025 [↑](#footnote-ref-1)
