# Supplementary material for: Can invitation systems increase participation in preventive health screening among adolescents?—an evaluation of a state-wide intervention in Germany using a difference-in-differences analysis of claims data
Source: Eur J Public Health. 2025 Mar 7;35(2):312–8. doi: 10.1093/eurpub/ckaf026 (PMC11967887; doi:10.1093/eurpub/ckaf026)
Supplement: ckaf026_Supplementary_Data [file ckaf026_supplementary_data.zip › ckaf026_Supplementary_Data/ejph-2024-11-om-0843-File005.docx]

Table S2. Stratified regression estimates of participation rates for J1, by education.

| Education | 1  lowest | 2 | 3  highest |
| --- | --- | --- | --- |
| Time | -0.0110^*^ (0.0045) | -0.0013 (0.0028) | 0.0006 (0.0032) |
| Treatment | 0.0096^***^ (0.0021) | -0.0278^***^ (0.0014) | -0.0282^***^ (0.0016) |
| Policy | 0.0180^**^ (0.0045) | 0.0055 (0.0028) | 0.0152^***^ (0.0032) |
| State fixed effects | **🗸** | **🗸** | **🗸** |
| C | 0.1989^***^ (0.0021) | 0.2459^***^ (0.0014) | 0.2642^***^ (0.0016) |
| *N* | 8100 | 122213 | 77183 |
| *R*^2^ | 0.001 | 0.001 | 0.001 |
| *adj. R*^2^ | -0.001 | 0.001 | 0.001 |

Standard errors in parentheses

^*^ *p* < 0.05, ^**^ *p* < 0.01, ^***^ *p* < 0.001
